# A Collaboratively-Derived Research Agenda for E-assessment in Undergraduate Mathematics

**DOI:** 10.1007/s40753-022-00189-6

**Published:** 2022-09-12

**Authors:** George Kinnear, Ian Jones, Chris Sangwin, Maryam Alarfaj, Ben Davies, Sam Fearn, Colin Foster, André Heck, Karen Henderson, Tim Hunt, Paola Iannone, Igor’ Kontorovich, Niclas Larson, Tim Lowe, John Christopher Meyer, Ann O’Shea, Peter Rowlett, Indunil Sikurajapathi, Thomas Wong

**Affiliations:** 1 School of Mathematics, The University of Edinburgh, Edinburgh, Scotland; 2 Loughborough University, Loughborough, England; 3 University College London, London, England; 4 Durham University, Durham, England; 5 University of Amsterdam, Amsterdam, Netherlands; 6 University of the West of England, Bristol, England; 7 The Open University, Milton Keynes, England; 8 The University of Auckland, Auckland, New Zealand; 9 University of Agder, Kristiansand, Norway; 10 Maynooth University, Maynooth, Ireland; 11 Sheffield Hallam University, Sheffield, England; 12 Heriot-Watt University, Edinburgh, Scotland; 13 University of Birmingham, Birmingham, England; 14 Saudi Electronic University, Riyadh, Saudi Arabia

**Keywords:** Online learning, Assessment, Feedback, Mathematics education, Delphi methods

## Abstract

This paper describes the collaborative development of an agenda for research on e-assessment in undergraduate mathematics. We built on an established approach to develop the agenda from the contributions of 22 mathematics education researchers, university teachers and learning technologists interested in this topic. The resulting set of 55 research questions are grouped into 5 broad themes: errors and feedback, student interactions with e-assessment, design and implementation choices, affordances offered by e-assessment tools, and mathematical skills. This agenda gives a framework for a programme of research aligned with practical concerns that will contribute to both theoretical and practical development.

## Introduction

Over the past two decades, e-assessment has become widespread in education, including for undergraduate mathematics. Here, we use the term *undergraduate mathematics* for the study of mathematics at the post-secondary level (for instance, in universities). Specialised e-assessment tools have been developed to meet the particular needs of assessing mathematics (Sangwin, 2013), for instance by allowing teachers to generate multiple randomised versions of a task, or by using a computer algebra system to check mathematical properties of students’ answers. Thus, uses of e-assessment in undergraduate mathematics range from employing general e-assessment tools in a mathematics context (e.g., uploading handwritten work, Jones & Alcock, 2014) through to use of mathematics-specific tools to implement what may be referred to elsewhere as *computer-aided assessment* or *computer-aided instruction* (e.g., Sangwin, 2013).

The increasing technical sophistication of e-assessment tools has underpinned their increased use in undergraduate mathematics (Iannone & Simpson, 2022). The Covid-19 pandemic has accelerated this trend: a survey of mathematics lecturers in the UK found that their use of e-assessment had further increased as a response to the pandemic (Alarfaj et al., 2022). Importantly, many lecturers planned to continue making use of such tools, “providing students with opportunities to practice, the ability to give immediate feedback, and the ability to randomise questions” (p. 69).

The widespread use of e-assessment in undergraduate mathematics presents a need for practitioners to be informed by existing research, and conversely for researchers to direct attention at emerging practical concerns. Much of the existing research on e-assessment is either not focused on the undergraduate level, or does not take account of the particular concerns of mathematics education. The importance of the topic for mathematics education researchers was confirmed by Bakker et al. (2021). Based on an international survey of mathematics education researchers, they identified “assessing online” as one of eight challenges for mathematics education research in the coming decade. They note the “significant advantages” over traditional approaches, but caution that “in an online environment it is even more challenging to successfully assess what we value rather than merely assessing what is relatively easy to assess” (p. 18).

What is needed is a programme of “use-inspired basic research” (Lester, 2005, p. 465), that contributes to both improved theoretical understanding and improved practice. To support such a programme of research, a research agenda can set out important questions that need to be addressed (e.g., English, 2008). Well-formed research questions thus play a crucial role in pursuing such a programme (e.g., Cai et al., 2019; Cai & Mamlok-Naaman, 2020).

In this paper, we report on a collaborative project that has established an agenda for such a programme of research. Our approach is inspired by a recent project to establish a research agenda in numerical cognition (Alcock et al., 2016), which was itself modelled on similar exercises in other fields (Sutherland et al., 2011). In the next section, we describe the process we used to achieve a similar outcome for e-assessment in undergraduate mathematics. In the remainder of the paper, we present the resulting set of 55 questions for future research in this area.

## Method

The project was initiated and led by Kinnear. A core team planned the project (Kinnear et al., 2020b), arranged and chaired meetings, and led the writing of this manuscript. Our plans were based on previous exercises that established research agendas in numerical cognition (Alcock et al., 2016) and conservation policy (Sutherland et al., 2011). The central idea is to use the Delphi method (Hsu & Sandford, 2007) to gather perspectives from a group of experts, then to work with the same expert group to refine ideas and develop consensus towards a research agenda. A hallmark of the Delphi method is an iterative approach; for instance, using experts’ responses to an initial questionnaire to design a survey, so that the whole group can give feedback on the range of contributions. We followed a five-stage process to develop the agenda, as described in the remainder of this section.

To recruit participants, we identified contacts from our existing networks, and from lists of participants at conferences focused on e-assessment for undergraduate mathematics. A key aim was to invite contributions from participants with a range of perspectives on the use of e-assessment in undergraduate mathematics, to include both theoretical and practical expertise. We generated an initial list of 22 contacts to invite beyond the core team, making sure that this list included a mix of education researchers, university teachers with experience of using e-assessment systems, and e-assessment system developers. Following suggestions from these contacts, we sent invitations (see Appendix A) to a further six people. This resulted in a group of $22$ participants, as shown in Table 1. Participants were predominantly in Europe and particularly the UK, reflecting the fact that the core research team is based in the UK.

**Table 1:** Participant locations, affiliations, roles (D = developer of e-assessment tools, R = mathematics education researcher, T = teaching mathematics using e-assessment), number of questions submitted at Stage 1, and number of questions edited at Stage 5. Note that some participants submitted no questions at Stage 1 since they joined later in the process

						**Questions submitted**	**Questions edited**
**Participant**	**Country**	**Affiliation**	**Roles**			**(Stage 1)**	**(Stage 5)**
André Heck	The Netherlands	University of Amsterdam		R	T	3	3
Ann O’Shea	Ireland	Maynooth University		R	T	3	3
Ben Davies	UK	University College London		R	T	2	4
Chris Sangwin	UK	University of Edinburgh	D	R	T	1	3
Colin Foster	UK	Loughborough University		R	T	3	5
George Kinnear	UK	University of Edinburgh		R	T	4	18
Ian Jones	UK	Loughborough University		R	T	5	10
Igor’ Kontorovich	New Zealand	The University of Auckland		R	T	3	5
Indunil Sikurajapathi	UK	University of the West of England		R	T	0	4
John Meyer	UK	University of Birmingham			T	0	2
Karen Henderson	UK	University of the West of England		R	T	5	4
Maryam Alarfaj	UK	University of Edinburgh		R		2	1
Morten Brekke	Norway	University of Agder			T	1	0
Niclas Larson	Norway	University of Agder		R	T	4	13
Paola Iannone	UK	Loughborough University		R	T	2	2
Pat Barmby	UK	No More Marking Ltd.	D	R		2	0
Peter Rowlett	UK	Sheffield Hallam University		R	T	9	26
Rhys Gwynllyw	UK	University of the West of England	D	R	T	4	0
Sam Fearn	UK	Durham University			T	0	1
Thomas Wong	UK	Heriot-Watt University			T	2	2
Tim Hunt	UK	The Open University	D			1	2
Tim Lowe	UK	The Open University			T	5	13

### Stage 1: Gathering Questions

We used an online survey to gather an initial set of candidate research questions. Participants were advised that questions should focus on formative or summative assessment of undergraduate mathematics that relies on technology in a fundamental way. We also asked participants to provide a short motivation for each question, and to describe what an answer might look like. This enabled participants to put the question in context, since “the significance of a research question cannot be determined just by reading it. Rather, its significance stands in relation to the knowledge of the field” (Cai et al., 2019, p. 118).

Participants proposed a total of 61 questions, with between 1 and 9 questions per participant and a mode of 2.

### Stage 2: Prioritising

Following Alcock et al. (2016), we planned to use an online rating system to identify the questions deemed most important by participants. However, as we had a relatively small ratio of questions (61) to participants (22), we decided this stage was not necessary, and we retained all of the questions.

### Stage 3: Refining Questions

We held a working group meeting, originally planned to be in-person but forced online by the Covid-19 pandemic, in which participants discussed the questions in order to suggest refinements and to identify connections between them. Prior to the meeting, the core research team divided participants and their proposed research questions into three groups. The groups were constituted so that each was chaired by one member of the core research team, and each group covered a range of countries, affiliations and interests. Participants were then asked to prepare for the meeting by reading in detail all the questions from their assigned group, and also browsing the questions from other groups to note any connections (see Appendix B for details).

The online meeting lasted two hours and was attended by $21$ of the participants listed in Table 1. During the meeting, the participants worked within their groups, with each group facilitated by a member of the core project team, and were asked to do the following:

- Clarify the questions by adjusting wording, or removing/combining/splitting questions as needed.
- Identify connections between questions and discuss possible themes.

Some participants also submitted written comments on the questions before or after the meeting.

Following the meeting, the core team met to consolidate the suggested refinements. Some of the original questions were deleted, while others were split into two or even three distinct questions. In refining the questions, we were guided by the “selection and refinement” criteria used by (Alcock et al., 2016, pp. 24–25), which were in turn drawn from Sutherland et al. (2011); for instance, that questions should “address an important gap in knowledge” and “be answerable through a realistic research design”. In addition, we paid particular attention to whether the questions were sufficiently within our scope, in that they were specific to the use of e-assessment within mathematics education.

This resulted in 52 questions. So that these could be presented in a structured way to a wider audience in the following stage, the core team arranged the questions into five sets of approximately equal size, using the connections identified by participants to try to keep related questions in the same set. These sets formed a basis for, but were ultimately different from, the themes presented later in this manuscript.

### Stage 4: Gathering Feedback

The activities described for Stage 3 were repeated, except for the preparation work, at three online conferences (E-Assessment in Mathematical Sciences, June 2020; Mathematics Education in the Digital Age, September 2020; 4th Northestern RUME Conference, October 2020). At each conference, the core team presented an overview of the project activities, before sharing the sets of questions and inviting conference participants to leave feedback using an online survey. This enabled input from a broader range of people (including internationally) in order to increase the validity and buy-in from the wider community.

At this stage, we focused on refining the existing questions, rather than adding more questions, although we invited survey respondents to suggest where we might have gaps. The core team reviewed the proposed new questions. Of the seven questions that were proposed, four were deemed to duplicate existing questions. The remaining three questions were included for the following stage.

### Stage 5: Finalising the Agenda

We planned to use conference participants’ feedback from Stage 4 to further focus the agenda. However, few of the conference participants completed the online survey. The responses we received indicated priorities that were widely distributed across the questions. As a result, we decided against removing questions of lowest priority from the list of questions, and instead, we retained the full set of 55 questions.

We invited the working group to populate a website for the project (https://maths.github.io/e-assessment-research-agenda/) that would host details about each question, including the motivation for the question, links to existing research and to other questions in the agenda, and possible approaches to answering the question. Most participants in Table 1 attended a 2-hour online meeting in January 2021 to begin the process of populating the website, following the guidance shown in Appendix C. During this meeting, small discussion groups formed organically to discuss questions related to particular topics, and to start making plans for future collaboration on those questions. Editing of the website continued asynchronously over the following months alongside preparation of this manuscript. Members of the working group added their names against those questions that they contributed to fleshing out on the website, with the number of questions for each member shown in Table 1.

Finally, we developed a structure to make the agenda easier to navigate (both in this manuscript and on the website). We used a thematic analysis approach (Braun & Clarke, 2006) to identify clusters of related questions, and to group those clusters into broad themes. The clusters and themes were initially formed by Kinnear, drawing on all the details about the questions on the project website to identify relationships between the questions. Minor adjustments to the clusters and themes were made through discussion among the core team during the process of finalising this manuscript.

## Research Agenda for E-assessment in Undergraduate Mathematics

The $55$ questions in the research agenda are arranged here in clusters of up to five tightly related questions, and these clusters are grouped into five broad themes. We emphasise that these themes are for coherence of presentation and do not reflect a hierarchy or intended prioritisation of the research questions. Alternative groupings of the questions would be possible, and we return to this issue in “Discussion”.

We present each theme in turn, and for each cluster of questions we provide a brief narrative to give a flavour of the details that are available on the project website (https://maths.github.io/e-assessment-research-agenda/). In particular, we expand on the intent of the questions, comment on possible motivations behind them, and highlight connections to existing research.

Before presenting the research questions, we note some decisions on consistent use of terminology throughout the manuscript. As noted in the introduction, we use *e-assessment* broadly to mean the use of computer technology to deliver assessment, whether this employs mathematics-specific tools or not. We use *teacher* or *lecturer* to refer to the person making decisions about the use of e-assessment as part of teaching, and *task designer* to refer to the person creating e-assessment tasks. The roles involved in designing and implementing e-assessment tasks are not always neatly separated. The task designer could be the lecturer or may be a distinct role for a learning technologist or even a student intern.

### Errors and Feedback

One of the most readily-identifiable themes is around detecting and responding to student errors. Feedback has long been identified as an educationally important intervention (Kluger & DeNisi, 1996), and there is advice in the general education literature on how to maximise its effectiveness (Shute, 2008). Many e-assessment systems can provide automated feedback, which responds to students’ answers in a way that is intended to improve their performance on the task (Sangwin, 2013). A wide range of features can be varied, such as the timing and content of feedback. Scholars have been investigating these variations (e.g., Attali & van der Kleij, 2017), but many questions remain unresolved.

#### Student Errors

1. *What common errors do students make when answering online assessment questions?*
2. *Do the errors students make in e-assessments differ from those they make in paper-based assessments?*
3. *What are the approaches to detecting and feeding back on students’ errors?*

Mathematics education has a well-established research tradition of analysing students’ work for common errors (e.g., Hart, 1987). The findings are then used to develop our understanding of mathematical learning, and to improve pedagogy and resource design. Question 1 invites a similar approach—and, since e-assessment systems typically store all student responses, there is a large set of historical and continuously generated data to be mined worldwide. Researchers working with e-assessment are therefore currently well-positioned to contribute to the literature on student errors (e.g., Sikurajapathi et al., 2020).

The validity and scope of such research would require addressing Question 2 to identify errors that arise specifically in e-assessments as opposed to paper-based assessments. In the former, there is often no requirement for students to show their solution method, and student errors can arise due to technology in ways that we would not expect in paper-based assessments (e.g., clicking the wrong button, or mistakes in typed syntax). We might hope that students routinely have a calculator, pencil and paper at hand, but this needs to be investigated (see also Question 13).

Question 3 relates to and precedes the research questions in “Feedback Design”. For example, upon detecting an error, we might need to discern whether it appears to have arisen from a common student error for the particular mathematical topic, or due to other reasons such as a misreading of the question or a miscalculation. This could be a challenging task when the e-assessment system has been used to generate different randomised variants of the same question.

#### Feedback Design

4. *How can content-specific features of provided feedback, for instance explanations with examples versus generic explanations, support students’ learning?*
5. *What are the linguistic features of feedback that help students engage with and use feedback in an online mathematical task at hand and in future mathematical activities?*
6. *What difficulties appear when designing e-assessment tasks that give constructive feedback to students?*

The design of feedback is a longstanding concern, both practically and for researchers. This group of questions addresses design issues highlighted by reviews of existing research on formative feedback (e.g., Shute, 2008; Palm et al., 2017).

Two of the questions have a particular focus on the design of elaborated feedback. A meta-analysis of different approaches to feedback in e-assessment observed that elaborated feedback existed on a “continuum ranging from very subtle to highly specific guidance” (Van der Kleij et al., 2015, p. 497), and noted that more research was needed to investigate the most effective approaches. Question 4 highlights a specific concern about the content of elaborated feedback: whether it should be based on generic explanations or should employ particular examples. For instance, in a question about adding fractions, the feedback could describe in general terms the process (“find a common denominator”, etc.) or could present a particular worked example. The two approaches may differ in the way they support students’ learning. Question 5 addresses the linguistic features of elaborated feedback, such as the appropriate level of detail and technical language to use. Investigation of this question may involve experimental comparisons of different approaches, but could also build on existing qualitative work that explored students’ interpretations of written feedback on proofs (Byrne et al., 2018).

More generally, Question 6 is concerned with exploring features that make feedback more constructive. The focus is on the difficulties encountered by task designers in preparing such feedback (this is related to questions about task design in “Design and Implementation Choices”). One feature that might be considered here is the distinction between explicit and implicit feedback (Devlin, 2011). For example, a question asking for a quadratic with given roots could produce an explicit message saying whether the student’s answer is right or wrong, or a graph of the function supplied by the student that implicitly conveys whether the answer is correct.

#### Emulating Teacher Feedback

7. *How can feedback that is dynamically tailored to the student’s level of mathematical expertise help a student use feedback on mathematical tasks effectively?*
8. *How useful for students’ long-term learning is feedback that gives a series of follow-up questions, from a decision tree, versus a single terminal piece of feedback?*
9. *What are the relative benefits of e-assessment giving feedback on a student’s set of responses (e.g. “two of these answers are wrong – find which ones and correct them”), rather than individual responses separately?*

The questions in this group are all motivated by a desire to use e-assessment to emulate features of teachers’ approaches. Despite the fact that previous researchers and developers working on intelligent tutoring systems “totally abandoned our original conception of [automatic] tutoring as human emulation” (Anderson et al., 1995, p. 202), it is likely that there are some aspects of the human teacher’s approach to feedback that could be fruitful in e-assessment.

Prior knowledge about students can influence the approach to giving feedback, and Question 7 asks how e-assessment might tailor feedback to best effect. An underlying motivation for this question is the expertise reversal effect (Kalyuga et al., 2003), which suggests that the relative effectiveness of different types of feedback may depend on a student’s level of task-specific expertise.

Question 8 asks whether a more dialogic approach to feedback could be effective in e-assessment. The suggestion is that, at each stage, the feedback given to the student should be the minimum that is necessary to direct their attention toward productive thinking about the task (i.e., a scaffolding and fading approach; see Mason, 2000; Foster, 2014). Similarly, Question 9 considers the possibility that making feedback deliberately less specific may be advantageous, as it may encourage students “to look at the feedback more closely and to think about their original work more analytically” (Wiliam, 2016, p. 14).

#### Optimising Feedback Efforts

10. *Under what circumstances is diagnosing errors worth the extra effort, as compared with generally addressing errors known to be typical?*
11. *What are the relative merits of addressing student errors up-front in the teaching compared with using e-assessment to detect and give feedback on errors after they are made?*
12. *In what circumstances is instant feedback from automated marking preferable to marking by hand?*

These questions are all motivated by practical concerns about how best to expend effort, particularly given that the effort required to detect and give appropriate feedback on specific errors may be nontrivial (see “Student Errors”). Question 10 asks whether it may be more efficient to give only generic feedback, such as a model solution. There are conflicting findings on this point in the literature (e.g., Rønning, 2017; Attali & van der Kleij, 2017), so the focus of this question is on identifying circumstances where the diagnostic effort is worthwhile.

In a similar vein, Question 11 asks whether the effort used for diagnosis and feedback could be better spent on developing up-front teaching that addresses common student errors. Foster et al. (2021, p. 12) characterise this distinction as “diagnose and treat” versus “treat all”, and favour the “treat all” approach in their work on curriculum design. Finally, Question 12 addresses a tension between immediacy and quality of feedback. Instant feedback is often argued to be beneficial because it corrects any errors while the student is still thinking about the question; however, it has also been noted that automated feedback may not be sufficiently responsive to students’ learning needs (Broughton et al., 2017; Rønning, 2017).

### Student Interactions with E-assessment

Many of the research questions that arose from this project can be related to students’ interactions with e-assessment. The questions in this group have an explicit concern with how students perceive and respond to e-assessments. We know from the general education literature that there is a link between students’ perceptions of the demands of learning and their engagement with learning (Marton & Säljö, 1997), and that interactions between students can play an important role. Accordingly, the questions straddle aspects of sociocultural, cognitive and design issues.

#### Student Behaviour

13. *How do students interact with an e-assessment system?*
14. *To what extent does repeated practice on randomised e-assessment tasks encourage mathematics students to discover deep links between ideas?*
15. *How do students engage with automated feedback? What differences (if any) can be identified with how they would respond to feedback from a teacher?*
16. *What should students be encouraged to do following success in e-assessment?*

These questions are all concerned with developing a better understanding of the impact that e-assessment has on students’ behaviour and approaches to learning. There are clear parallels with questions about errors and feedback in “Errors and Feedback”, and with questions about task design in “Design and Implementation Choices”, but the questions in this group offer a different perspective. Rather than being focused on details of the design choices facing task designers, these questions are concerned with the implications of those choices for how students engage with e-assessment and their studies more generally. Thus, the questions have a similar motivation to work on understanding how students respond to other aspects of teaching, such as the use of lecture recordings (Wood et al., 2021; Lindsay & Evans, 2021) or guided notes (Iannone & Miller, 2019).

Question 13 is expressed in general terms and some relevant studies have already been carried out to investigate students’ behaviour. For instance, Dorko (2020) used video recordings of students undertaking online homework along with follow-up interviews and reported that feedback and multiple attempts offered by the online system contributed to students working iteratively when solving problems. Further research should investigate the robustness of Dorko ’s findings across different technologies, cohorts and contexts. Other aspects of student behaviour, such as social interactions when using e-assessment systems, should also be considered (see “Student Interactions”).

Question 14 considers the effect of repeated practice on students’ behaviour; specifically, whether students are led to develop understanding of underlying structures through engaging with randomised versions of a task, or whether they are led merely to “pattern spotting”. This is relevant to current work in applying cognitive science to mathematics practice, such as interleaving (Rohrer et al., 2015) and spacing (Lyle et al., 2020).

The final two questions are concerned with how students make use of feedback from an e-assessment system. This clearly has connections with many of the previous questions about feedback (discussed in “Errors and Feedback”), but here the focus is on how students behave in response. Question 15 highlights that students may respond differently to the same feedback if it were given by a teacher. This is informed by previous research noting differences in student behaviour that could be attributed to the e-assessment system being machine-based rather than human (Jordan, 2012). Question 16 is motivated by the observation that, for some students, attaining a desired result in an assessed task can result in disengagement from further learning. This can be a particular concern in courses where the e-assessment tasks are designed to cover only some of the desired learning outcomes; for instance, the more procedural aspects. Thus, work to address this question should take account of the way e-assessment is used in course design (see related questions in “Role of E-assessment in Course Design”).

#### Student Views and Outcomes

17. *What are students’ views on e-assessment, and what are their expectations from automated feedback?*
18. *How might dyslexic, dyscalculic and other groups of students be disadvantaged by online assessments rather than paper-based assessments?*

These questions take a broader view of students’ interaction with e-assessment than those in the previous section.

Question 17 asks about students’ views on e-assessment, and of automated feedback in particular. In a review of existing literature on student perceptions of feedback, Van der Kleij and Lipnevich (2020) considered 164 studies. However, only four of these were based in mathematics, and a similarly small number were related to automated feedback. The way that e-assessment is used in a course will clearly be an important factor influencing students’ views toward it; thus, answers to this question should pay careful attention to the context. For instance, Rønning (2017) found that where e-assessments were compulsory, and unlimited attempts were allowed, students viewed the e-assessment as a process of “hunting for the answer”. It would also be worthwhile to understand students’ views in relation to other forms of assessment, particularly given that mathematics undergraduates tend to express a preference for more traditional forms of assessment (Iannone & Simpson, 2015).

Question 18 is concerned with the potential for differential outcomes for various groups of students, based on their use of e-assessment. For instance, it could be the case that dyslexic students face additional barriers when using e-assessment, and therefore perform less well than might be expected. There is a notable lack of research on this topic, especially given the increasingly widespread use of e-assessment. Indeed, (Cai et al., 2020, p. 525) noted that “an important question for the field is how to prevent technology from reproducing or even widening the inequities in learning opportunities across groups of students”. Question 18 highlights students with specific learning difficulties as a particular group to consider. However, the intended scope is broader since inequalities could arise in other ways, such as accessibility challenges (e.g., vision impairment), or from working in a non-native language.

#### Student Interactions

19. *How can peer assessment be used as part of e-assessment?*
20. *How can e-assessment be used in group work, and what effect does the group element have on individuals’ learning?*

Peer assessment is trumpeted for potential benefits such as generating more student feedback than can be given by a lecturer (Topping, 2009), and promoting learning through students viewing one anther’s work (Jones & Alcock, 2014). Research on peer assessment in higher education increasingly involves technology (e.g., Ashenafi, 2017), although mathematics-specific e-assessment technologies tend not to overtly support peer learning and assessment activities. Studies addressing Question 19 are likely to focus on generic peer assessment technologies that can be used for mathematics (such as comparative judgement, as discussed in “Comparative Judgement”).

Nevertheless, students sometimes spontaneously form support groups when taking online tests (Alcock et al., 2020). Lecturers may wish to encourage students to work in groups to harness the benefits of peer learning. Question 20 relates to how understanding the way students collaborate when working on e-assessments can help us better understand how to promote collaborations that are productive for learning.

### Design and Implementation Choices

The questions in this group are primarily concerned with the choices that must be made by the lecturer or task designer, whether at the level of designing an individual e-assessment question, or at the level of integrating e-assessment into a coherent course design.

#### Task Design Principles

21. *What design methodologies and principles are used by e-assessment task designers?*
22. *What principles should inform the design of e-assessment tasks?*
23. *E-assessment task designers often convert questions that could be asked on a traditional pen and paper exam: what are the implications, technicalities, affordances and drawbacks of this approach?*

There is a large body of work on the design of mathematics tasks, motivated by the influence that tasks can have on students’ learning (Breen & O’Shea, 2019). The design of e-assessment tasks can be informed by this previous work, but there are additional considerations that are specific to e-assessment which warrant further study. Design principles are often implicit in task designers’ practice, and Question 21 seeks to make them explicit. Examples of design principles being made explicit in previous work include Sangwin's (2013, Chapter 3) description of general principles of assessment design, and Kinnear et al.'s (2021) account of the specific principles that informed the design of an online course built around e-assessment. An answer to Question 21 could be based on a systematic review of such literature. It may also be informed by work on Question 23, which highlights the common practice of converting or “translating” existing paper-based tasks to e-assessment. This approach may have implications for the range of tasks that are used in e-assessment—some existing paper-based tasks may be “untranslatable”, while other tasks that would be suited to e-assessment may not be considered.

Question 22 goes beyond asking what principles are currently in use, and seeks to identify the principles that *should* be used. This could be informed by work on Question 21 (and others, e.g., from “Errors and Feedback”) to identify possibilities, as well as drawing on the expertise of e-assessment task designers.

#### Randomisation

24. *To what extent does the randomisation of question parameters, which makes sharing answers between students difficult, adequately address plagiarism?*
25. *What effect does the use of random versions of a question (e.g., using parameterised values) have on the outcomes of e-assessment?*
26. *When writing multiple choice questions, is student learning better enhanced using distractors based on common errors, or randomly-generated distractors?*

The ability to randomly generate versions of questions is a core feature of many mathematics e-assessment systems and “creates opportunities not present with a fixed paper worksheet” (Sangwin, 2003, p. 38). One of the justifications given for the importance of generating different versions of a question for each student is that it mitigates against plagiarism. Question 24 asks whether this is really the case, and suggests replicating and extending quantitative work (e.g., Arnold, 2016) that seeks to detect the possible extent of plagiarism, with a particular focus on whether the use of randomisation reduces it.

Question 25 asks about the effect of randomisation on outcomes more generally. A particular concern here is the effort required to devise and test randomised questions, and whether this is justified by the suggested benefits (such as giving students the opportunity for repeated practice of key skills). The issue of authoring effort is also relevant in Question 26, which focuses on multiple choice questions. A standard recommendation to authors of multiple choice questions is to base distractors on common student errors (e.g., Gierl et al., 2017). Given the difficulty that task designers face in anticipating such student errors (see “Student Errors”), it could be more efficient to use randomly-generated distractors for questions with numerical answers; there is also the possibility that students who make an error could benefit from what is effectively immediate feedback when they do not see their answer listed as an option.

#### Role of E-assessment in Course Design

27. *How can formative e-assessments improve students’ performance in later assessments?*
28. *How can regular summative e-assessments support learning?*
29. *What are suitable roles for e-assessment in formative and summative assessment?*
30. *To what extent does the timing and frequency of e-assessments during a course affect student learning? *
31. *What are the relations between the mode of course instruction and students’ performance and activity in e-assessment?*

These questions are all concerned with decisions about how e-assessment can be used within a course, such as the timing and incentives associated with completing the assessments.

One important course design decision is whether e-assessments are used formatively or summatively. Formative e-assessments are commonly used to provide students with opportunities to practise skills, consistent with the view that “mathematics needs to be *done* to be *learned*” (Greenhow, 2015, p. 120, emphasis in original). However, the extent to which students engage with these formative assessments is variable and, in some cases, time spent by students on e-assessment can be detrimental to their overall performance (Hannah et al., 2014). Thus, Question 27 is concerned with identifying ways that e-assessment can be employed formatively to best effect. Summative e-assessments can range from a small portion of the course grade, through to forming the basis of the entire course (e.g., Sangwin & Kinnear, 2022). Question 28 asks how such summative uses can support learning, and the suggested outcome is a collection of case studies of different models, together with some evaluation of their effectiveness.

Question 29 ties together Questions 27 and 28, and asks how both formative and summative assessments can be used as part of course design. Each approach may be suitable for achieving different aims, and this is closely related to the affordances of the e-assessment tool (see also “Affordances Offered by E-assessment Tools”). For instance, current tools are perhaps best suited to assessment of procedural skills, and this informs the ways that e-assessment can be used as part of course design.

Another course design decision concerns the frequency and timing of e-assessment. For instance, some courses make extensive use of e-assessment with weekly (or even more frequent) tasks for students to complete (e.g., Kinnear et al., 2021; Heck et al., in press). Question 30 invites a comparison of different approaches, such as between the use of shorter more frequent e-assessments throughout a course, and fewer e-assessments (e.g., at the end of substantial topics).

Finally, Question 31 seeks to understand how different approaches to course design influence the way that students interact with, and perform in, e-assessment as part of the course. For instance, there is now a large body of research showing that active learning approaches are more effective than traditional lecturing (Freeman et al., 2014), including studies of mathematics teaching (e.g., Maciejewski, 2015). E-assessment has not been prominent in this research so far.

#### Lecturer Guidance

32. *What advice and guidance (both practical and pedagogical) is available to lecturers about using e-assessment in their courses, and to what extent do they engage with it?*
33. *What might a “hierarchy of needs” look like for lecturers who are transitioning to increased use of e-assessments?*
34. *How can lecturers be informed about how students interact with e-assessment tasks, and so help lecturers act upon these findings in an effective way?*

Moving from analysis of student behaviours (see “Student Behaviour”) to implications for practitioners is not straightforward (Alcock et al., 2020), and addressing these research questions might require a programme of research projects.

Question 32 arose from a context in which lecturers had to create materials from scratch and devise questions of their own. Analysis of such materials, along with methods such as interviewing the authors of the materials, could be informative. Question 33 might be answered using the same methods and case studies, with analysis focusing on identifying the steps, skills, equipment, expertise and so on required to construct e-assessments. This could build on existing guidance on task design for undergraduate mathematics (e.g., Breen & O’Shea, 2019), by addressing the additional considerations that are required in e-assessment.

Question 34 focuses not on *a priori* design principles, but on monitoring and responding to students’ interactions. Some e-assessment systems offer data on students’ engagement with them; for example, systems such as STACK or SOWISO provide data on student responses and results (Sangwin, 2013; Rienties et al., 2019). However, the data can be overwhelming for lecturers, so it is not always clear how they should act in response. Olsher et al. (2016) present an example showing the promise of distilling students’ e-assessment response data so that it might inform subsequent teaching, and further work is needed to make such approaches more attainable.

### Affordances Offered by E-assessment Tools

These questions are about the features of existing e-assessment tools and the relationships of these tools to assessment objectives. The research questions consider what is possible, the extent to which this fulfils various purposes, and ways in which the features could be extended. There is a subtle interplay between the assessment format, questions we ask students, and the overall course design (as discussed in “Roles of E-assessment in Course Design”). This has always been the case and is not only an issue for e-assessment; for instance, paper-based multiple choice questions require careful design (Gierl et al., 2017) and using them to assess extended forms of reasoning is difficult.

#### Capabilities of E-assessment

35. *What types of reasoning are required to complete current e-assessments?*
36. *To what extent do existing e-assessments provide reliable measures of mathematical understanding, as might otherwise be measured by traditional exams?*
37. *How can e-assessment support take-home open-book examinations?*
38. *What developments at the forefront of e-assessment (such as artificial intelligence) can we apply to undergraduate mathematics?*

These questions, as a group, form a natural sequence, starting with the content of individual tasks (Question 35). Lithner (2008) developed a framework for classifying the types of reasoning required by mathematical tasks. Other similar task classification schemes were proposed by Smith et al. (1996) and Pointon and Sangwin (2003). Such classification schemes have been used for research (e.g., Darlington, 2014) and more informally as aids to the design of formative assessments. Such frameworks enable the study of the types of tasks assigned in undergraduate courses (Kinnear et al., 2020a; Mac an Bhaird et al., 2017); however, it seems that very little related work has been published in the area of e-assessment.

The next two questions relate to the capability of e-assessment to support, or even replace, traditional examinations (i.e., time-limited, closed-book and invigilated). Traditional examinations have a number of advantages. Examinations place a highly constrained time limit on the assessment (in a way more open project work does not), and lecturers can be reasonably confident that impersonation of the candidate does not take place. The controlled conditions within a traditional examination allow explicit choices to be enforced, such as the availability of books and other reference materials, and the use of technology such as calculators. There is also the social experience of the “event” when attending a traditional examination venue en-mass with peers, which may even be seen as a rite of passage. Notwithstanding criticism of traditional examinations, they remain an important baseline against which e-assessment may be judged.

Question 36 is concerned with how the results from e-assessment compare with examinations in terms of their reliability. A specific concern is the extent to which e-assessment can be used to measure mathematical understanding, considering the types of tasks that can be set using e-assessment (as in Question 35; see also “Mathematical Skills”). To address this concern, previous work has sought to replicate tasks from traditional examinations using e-assessment with automatic grading (e.g., Sangwin & Köcher, 2016; Sangwin, 2019). The proportion of tasks that can be faithfully replicated in this way, using current tools, is perhaps surprisingly high.

Question 37 asks about the role of e-assessment in supporting open-book exams. Many institutions turned to open-book exams due to the Covid-19 pandemic, albeit with concerns about potential for “academic integrity breaches” (Seaton et al., 2022, p. 562). The use of e-assessment to randomise values has been suggested as one way to address this concern (see also “Randomisation”). A process where randomised questions are generated by an e-assessment system, but the resultant submissions are marked by hand, is explored by Rowlett (2022). A hybrid approach, where part of the submission is marked automatically and passed to a human marker, may also be possible.

Finally, Question 38 looks to the future, and to how new developments in e-assessment technology can be applied in undergraduate mathematics. These developments include advances in interpreting free-form input (taken up further in “Free-form Student Input”), and the use of artificial intelligence to augment or replace human judgement in assessments.

#### Free-form Student Input

39. *What methods are available for student input of mathematics?*
40. *How can the suitability of e-assessment tools for summative assessment be improved by combining computer-marking and pen-marking?*
41. *Are there differences in performance on mathematics problems presented on paper versus as e-assessments?*
42. *How can we automate the assessment of work traditionally done using paper and pen?*
43. *How can we emulate human marking of students’ working, such as follow-on marking and partially correct marking?*

These questions relate to automatic assessment of complete mathematical arguments. Assessment of students’ free-form input probably remains one of the most significant challenges in e-assessment, both from the technical perspective of software design, and the complexity of the traditional assessment process.

A central problem is how students might input their response into a computer (Question 39). Most professional mathematicians typeset their work with systems like LaTeX. However, LaTeX can be time-consuming to learn, and might be too cumbersome for students to use to submit their answers (particularly for assessments that have a short time limit). There have been many attempts to provide students with constrained interfaces to help them work line-by-line, from MathXpert (Beeson, 1998) through to SOWISO (Heck, 2017). Another option is to photograph handwriting and upload it, perhaps in addition to keying in a final answer; this sort of hybrid approach is considered in Question 40. However, most of the questions tacitly assume moving beyond merely uploading an image.

One of the significant barriers faced by mathematics students interacting with e-assessment is the heavy use of special symbolism. This motivates Question 41, since the use of special syntax is one factor that may lead to a difference in performance in e-assessment compared with paper. For example, Sangwin and Ramsden (2007) found that a substantial number of student errors in one e-assessment system were due to difficulties in using the particular “linear syntax” for entering expressions. Notational ambiguity occurs between mathematical sub-disciplines, unsurprisingly leaving potential for serious confusion about the interpretation and meaning of students’ work (see, e.g., Kontorovich & Zazkis, 2017). All this said, e-assessment provides an opportunity to explore and research students’ intended meaning, precisely because the syntax is often rather strict.

Question 42 seeks to understand the extent to which we can automate the assessment of mathematical work traditionally done using paper and pen. In previous research, tasks from traditional examinations were successfully re-implemented using e-assessment with automatic grading (e.g., Sangwin & Köcher, 2016; Sangwin, 2019); however, the tasks were limited to assessing the students’ final answers. Further work is needed to consider students’ line-by-line working. Question 43 builds on this by considering the possibility of “follow-on” marks awarded for correct working after an error, or awarding partial marks (e.g., for correct use of a method in an intermediate step). Beyond replicating these traditional examination marking approaches, e-assessment unlocks the possibility of adaptivity. For instance, when an error is identified in a student’s answer, they could be shown some feedback and invited to correct their answer. Ashton et al. (2006) demonstrated such an approach, where students could achieve partial credit for completing a version of a task that had been broken down into steps.

#### Comparative Judgement

44. *How can comparative judgement be used for e-assessment?*
45. *How can e-assessment using comparative judgment support learning?*

Comparative judgement offers a method of grading students’ work that involves making holistic judgements about the relative quality of students’ work without reference to a rubric (Pollitt, 2012). These holistic decisions are made for numerous pairs of students’ work. The decisions are used to derive a score for each piece of student work, and these scores can then be used for ranking or grading purposes as required. Comparative judgement has received attention in mathematics education over the past decade due to its promise as a reliable and valid method for assessing important but nebulous learning outcomes, such as conceptual understanding (e.g., Jones & Alcock, 2014), proof comprehension (e.g., Davies et al., 2020), and problem solving (e.g., Jones & Inglis, 2015). Since comparative judgement enables the use of genuinely open-ended tasks, it represents an opportunity to broaden the assessment diet (Iannone & Simpson, 2022), beyond the traditional e-assessment focus on mathematical accuracy. Question 44 considers issues of using comparative judgement for assessment, whereas Question 45 considers student learning, although in practice both questions are likely to be intertwined and addressable through iterative design research methods (McKenney & Reeves, 2018).

The potential barriers to lecturers using comparative judgement for summative assessment include comparative judgement’s lack of rubrics, meaning students might not know what they are aiming for, and its lack of traditional ‘red ink’ feedback. For some higher education institutions, these aspects may be unacceptable. Nevertheless, comparative judgement can and has been implemented in higher education institutions (e.g., Jones & Sirl, 2017), and when used for formative assessment, the potential barriers for summative assessment can be strengths for student learning. For example, through structured peer assessment activities (see also Question 19 in “Design and Implementation Choices”), students, rather than lecturers, can judge one another’s responses to open-ended test questions to identify what constitutes a high-quality response in the absence of rubrics. Moreover, feedback can be reconceptualised in terms of students comparing one another’s responses with reference to their own, rather than in terms of ‘red ink’ received from the teacher or e-assessment system.

### Mathematical Skills

While the whole research agenda is specific to undergraduate mathematics, the questions in this theme are particularly directed at how e-assessment can be used to assess and develop particular mathematical skills: problem solving, proving and generating examples. Numerous works have discussed the nature of mathematics and the skills that mathematics education might seek to develop (e.g., Pólya, 1954; Freudenthal, 1973; Mason et al., 2010). E-assessment has, mostly, been used to provide formative feedback for procedural practice, often in calculus and algebra. Existing e-assessment tools, and contemporary know-how in using them, start to become less useful for the more challenging questions raised in this section. However, our experience suggests that e-assessment can be applied in some areas to good effect, and further work may identify opportunities and where the boundaries of effective e-assessment really lie.

#### Problem Solving

46. *How can we assess problem solving using e-assessment?*
47. *How can we assess open-ended tasks using e-assessment?*
48. *How can e-assessments provide scaffolding (cues, hints) during and after problem-solving tasks?*

These three questions are closely related. Answers to these questions have been among the goals of the earliest pioneers of e-assessment, who worked on what were often called *intelligent tutoring systems* or *computer-aided instruction*. By the 1980s, these rather ambitious goals were being reassessed (see Sleeman & Brown, 1982, Preface); however, such goals are still of practical interest.

One common approach to assessing problem solving (Question 46) has been to break up larger tasks into smaller individual questions to which e-assessment can then be applied. The e-assessment system then builds up a model of the state of the students’ knowledge (e.g., Appleby et al., 1997). The drawback of this approach is that it requires a significant investment of time and experience to develop a suitable question bank (Anderson et al., 1995).

Automatically assessing a genuinely open-ended task (Question 47) poses two main difficulties. First, the student must have a means to enter their answer in a machine-readable format, which itself is nontrivial (see “Free-form Student Input”). Second, automation requires “preemptive” (Sangwin, 2013, p. 35) decisions: the task designer must anticipate likely approaches from students and decide how they will be graded, before students have completed the task. Progress on this question could perhaps be achieved through cycles of design research, to develop prototypes of tasks, test them with students, and iteratively make improvements.

Question 48 is concerned with approaches to scaffolding the problem-solving process. One approach, illustrated by Beevers and Paterson (2003), allows students to opt to receive hints about how to proceed with a given problem. While this early work was focused on secondary school mathematics, there is ongoing development of tools to facilitate similar approaches at undergraduate level (e.g., Harjula et al., 2017).

#### Assessment of Proof

49. *How can the assessment of proof be automated?*
50. *What can automated theorem provers (e.g. LEAN) offer to the e-assessment of proof comprehension?*
51. *What types/forms of proof-comprehension-related questions can be meaningfully assessed using currently available e-assessment platforms?*
52. *How can students effectively type free-form proof for human marking online?*

Proof is the hallmark that distinguishes mathematics from other subjects (Hanna, 1983). However, the meaning of “assessment of proof” (Question 49) is open to interpretation and has a number of subtly interrelated aspects. Deciding when a proof is correct is far from easy, since teachers have differing expectations on the level of detail required. What constitutes an acceptable size of gap in the reasoning for the particular class differs significantly between subject areas; for instance, logic and foundation courses might require that students make much more specific reference to axioms and particular deduction rules than a traditional calculus methods course. Both contain proofs, but they look very different. Such variety is a significant challenge in general, although it is certainly possible to assess proof in particular constrained areas of mathematics using specially-designed tools (e.g., Gruttmann et al., 2008; Vajda, 2009; Vajda et al., 2009).

That said, mathematical proof may (or may not) be a more constrained and structured language than other subject areas. This is perhaps where automated theorem provers have a role, as considered in Question 50. By their very nature, automatic theorem proving software constrains users to the syntax and norms of the prover. This makes the proof checking possible, but also creates a significant barrier to use (Avigad, 2019). Learning to use the theorem prover becomes as much a part of the process as learning the mathematics that is to be proved (e.g., Thoma & Iannone, 2021). The use of theorem provers has not been widely accepted within many sub-disciplines of the mathematics community, and asking students to learn to use a theorem prover may not be an acceptable path for many colleagues. However, automated theorem provers are attracting some interest in teaching first-year students as a way to develop programming skills.

Question 52 is concerned with methods for student input of free-form proofs for human marking. Human marking removes the need for students to use the highly-constrained syntax of a theorem prover, but new input methods could introduce other (perhaps helpful) constraints. For instance, an input method could help students to construct proofs using “proof frameworks” (Selden et al., 2018), that emphasise the block structure of a proof. Of course, the overarching issues with free-form input would still need to be addressed; see “Free-form Student Input”.

One promising area in the short term is not assessment of free-form proofs, but rather assessment of aspects related to proofs (Question 51). A number of aspects were suggested, but not evaluated, by Sangwin and Bickerton (2021), including proof comprehension, which was discussed in detail by Mejia-Ramos et al. (2017). Another promising approach was taken by Davies et al. (2020): students were tasked with writing a short summary of a given proof, and comparative judgement (see “Comparative Jugdement”) was used for e-assessment.

#### Example Generation

53. *How can e-assessments be designed to expand and enrich students’ example spaces?*
54. *To what extent can e-assessments meaningfully judge student responses to example generation tasks?*
55. *How does the use of e-assessment impact students’ example generation strategies and success, relative to the same tasks on paper or orally?*

These questions allude to research suggesting that example generation tasks can encourage students to engage with new concepts, and to expand their awareness of representations and instances of those concepts (Watson & Mason, 2006). However, feeding back on student-generated examples can be a difficult task for teachers, and is impractical with large cohorts. As such, e-assessment could play a powerful role in providing students with formative feedback on their examples (Sangwin, 2003).

Questions 53 and 54 might involve investigating how existing, paper-based tasks can be implemented in e-assessment systems and, importantly, how we can reliably check the properties of students’ inputted examples. For example, if asking for functions which tend to 0 at infinity, the e-assessment system would need to have the ability to evaluate limits of arbitrary functions. Further limitations to automating the feedback of example generation tasks are likely to be encountered; for example, students may immediately be able to sketch an example with the required properties, or orally describe the relevant features, but it is not obvious how such responses can be inputted in a format that would be amenable to automated feedback. Given the difficulty of accepting free-form input (as discussed in “Free-form Student Input”), existing tasks often use constrained interfaces, e.g. presenting a quadrilateral and allowing the points to be dragged to new positions (Popper & Yerushalmy, 2021). Question 55 is concerned with evaluating the educational benefits of such approaches, using paper-based or oral example generation tasks as a control.

## Discussion

Through a collaborative process, we have developed a shared agenda for research on e-assessment in undergraduate mathematics. The agenda consists of 55 research questions that we have grouped into 5 broad themes: errors and feedback, student interactions with e-assessment, design and implementation choices, affordances offered by e-assessment tools, and mathematical skills. The wide range of questions underscores the complexity of research on learning, with topics ranging from issues of design and implementation, through to cognitive, social, and sociocultural issues. Research to advance this agenda will therefore benefit from drawing on a range of theoretical perspectives (Lester, 2005).

The agenda’s broad range of questions invites a range of research approaches. Some of the questions could be addressed by literature reviews or analysis of existing data. For instance, methodologies and principles used by e-assessment task designers (Question 21) could be identified by reviewing literature (e.g., Greenhow, 2015; Sangwin, 2013; Sangwin & Bickerton, 2021; Heck et al., in press). Other questions would benefit from qualitative approaches, such as interviewing students to better understand their views on e-assessment (Question 17), or observing students as they work on tasks to gain insight into their processes (e.g., example generation strategies in Question 55). Many of the questions would be best addressed using experimental methods, which have been relatively under-used in the field of mathematics education in recent years (Alcock et al., 2013; Inglis & Foster, 2018). For instance, questions about novel approaches to feedback (“Emulating Teacher Feedback”) could naturally be addressed with randomised controlled trials comparing student outcomes under different feedback conditions. Moreover, some questions could be suitable for a multi-site approach, similar to the recent *ManyClasses* study on the efficacy of delayed feedback (Fyfe et al., 2021). For instance, comparing different approaches to timing of assessments (Question 30) across many contexts could enable a better understanding of possible factors influencing their effectiveness.

We have not made any attempt to prioritise questions in the agenda. Previous exercises have included a round of prioritising to reduce the original list of questions submitted by participants to those deemed most important (e.g., Alcock et al., 2016; Sutherland et al., 2011). While we had originally planned to do this at Stage 2, we decided that this was not necessary since the set of submitted questions was of a manageable size. Instead, we simply consolidated the few submissions that overlapped. We believe this is a pragmatic approach that has enabled the agenda to address a diverse range of concerns.

While we have presented the questions here arranged into five themes, the themes are simply a tool to enable a coherent presentation of the agenda (as noted at the start of “Research Agenda for E-assessment in Undergraduate Mathematics”). Other ways of categorising or grouping the questions are possible. For instance, during the initial planning of the project, we anticipated a possible grouping based on different levels of generality (Kinnear et al., 2020b, p. 379); we decided not to pursue this, in favour of a bottom-up approach. It would also be possible to consider separately those questions that are strongly related to mathematics-specific e-assessment tools, and those that employ general tools in a mathematics context. Alternatively, the questions could be grouped according to possible research approaches: for instance, many of the questions take the form “How can...?” and accordingly may benefit from expertise in design research.

The research agenda reflects the interests and concerns of our working group. This could mean that the agenda is not representative of the wider field. For instance, our group is predominantly based in the UK, and many of us share a background in working with a particular set of e-assessment tools. However, our approach of presenting the agenda at various international meetings provided us with feedback on the emerging agenda from different perspectives, and the questions are not specific to any particular platform. Nevertheless, the agenda may lack emphasis on priorities that arise in other contexts. For instance, many undergraduate classes in the UK are large, with lecturers responsible for setting assessments for hundreds of students. Large classes clearly lead to some different priorities for e-assessment than in systems with smaller class sizes, which is perhaps implicit in many of the questions about using e-assessment to “scale up” teaching approaches, e.g. “Emulating Teacher Feedback”. Another example of priorities we may not have captured comes from recent work to introduce e-assessment with large undergraduate classes in Kenya, which noted that “though most of the students do not have laptop computers, they were able to access the quizzes through their smart phones” (Oyengo et al., 2021, p. 7). This issue is not something our group discussed, and may have inspired further questions, e.g. in “Free-form Student Input”.

While we do not claim that our agenda is universal, it is clear that many of our concerns are shared by other researchers (e.g., Bakker et al., 2021; Cai et al., 2020). One notable omission that became apparent near the end of our process is a lack of questions related to diversity, equity and inclusion. While we have one question in this vein (Question 18), there are clearly other important questions that could have been included. For instance, one of the participants in the survey by Bakker et al. (2021) asked: “What roles could digital technology play, and in what forms, in restoring justice and celebrating diversity?” This question could be grounded in the context of e-assessment as well.

One of our aims in carrying out this exercise was to stimulate new research collaborations to address these questions. This has already had some success: new collaborations are underway, addressing various questions from the agenda. Members of the group would welcome further collaboration with the wider community. To facilitate this, the project website (https://maths.github.io/e-assessment-research-agenda/) shows which group members have particular interest in each of the questions. This site will be updated as progress is made on addressing the questions.

We also aimed to offer a method to the undergraduate mathematics education community, in which researchers and developers often overlap and work together, for setting research agendas. The focus here has been specific – e-assessment in undergraduate mathematics – but the method could readily be applied to other topics, or indeed to education research and development more widely. We would recommend the use of a collaborative repository to gather and track input from participants. We introduced this during Stage 5 of our process, and in retrospect would have benefited from using this from the beginning to encourage participants to share more details earlier on in the process. We would also encourage future projects adopting this method to pay particular attention to the range of participants involved, to ensure that a broad range of perspectives are included.

## A. Invitation Email

I am writing to invite you to collaborate on a new working group that aims to develop a shared research agenda for computer-aided assessment of undergraduate mathematics. Such an agenda will help to establish a programme of research aligned with practical concerns, which would contribute to both theoretical and practical development. The working group is being run by George Kinnear, Chris Sangwin and Ian Jones.

We will follow methods used to develop a research agenda in numerical cognition, see Alcock et al. (2016).

We will begin with an online phase to gather and prioritise an initial set of questions. This will be followed by a series of in-person meetings to discuss and further prioritise the questions. Throughout the process input from the broader research community all also be sought. All collaborators will be invited to contribute to a scientific paper to publish the agenda in a peer reviewed outlet. For reference, I’ve attached a short paper that we’ve written explaining the project in more detail. *[the attachment was a copy of Kinnear et al.* (2020b)*]*

At this stage we are seeking three things from you. First, a reply to this email to let us know if you are interested or not. Second, suggestions for colleagues we could also contact. Third, completion of an online survey to gather the initial set of questions as described above. The survey can be found at https://edinburgh.onlinesurveys.ac.uk/questions-in-online-assessment

We hope you are able to give serious consideration to this request. We’d love to have you on board!

## B. Instructions Ahead of Stage 3 Meetings

- “Read all the questions from your group. Note any queries or comments you can bring to the discussion for instance:
  - ◦ are there statements that could be clarified?
  - ◦ do you know of any existing related research?
  - ◦ do you have thoughts on the possible approaches to answering the question?
- Look at questions from the other groups and note any connections with the questions that you proposed. This will speed up the process of us identifying overlapping concerns, while distributing the effort.”

## C. Instructions on Editing Questions

This repository gives us a way to collaborate on writing about the set of research questions.

As a contributor, you should:

1. make sure you are happy with your profile,
2. add yourself as a contributor on any questions you are interested in,
3. edit the pages of those questions, to add/amend details.

Most questions only have the text of the question so far. Please help to flesh out the details!

This can be informed by:

- the original submission – see the document from our first meeting which has the original proposer’s full narrative,
- comments from the survey/conferences – you can see all of these in the files in Google Drive,
- your own further thoughts/reading.

Adding related questions.

Many of the questions are connected, and we’d like to highlight this. There is a section on each question page for “Related questions” which should end up with a list of points about how the question connects with others.
